# A Retrospective Study on the Risk of Respiratory Distress Syndrome in Singleton Pregnancies with Preterm Premature Rupture of Membranes between 24+0 and 36+6 Weeks, Using Regression Analysis for Various Factors

**DOI:** 10.1155/2018/7162478

**Published:** 2018-10-04

**Authors:** Anna Niesłuchowska-Hoxha, Wojciech Cnota, Bartosz Czuba, Aleksandra Ruci, Magdalena Ciaciura-Jarno, Agnieszka Jagielska, Dominik Wójtowicz, Rafał Kierach, Krzysztof Dąbrowski, Marcin Sidorowicz, Wioletta Skrzypulec-Plinta, Agata Wloch, Dariusz Borowski, Piotr Węgrzyn

**Affiliations:** ^1^Department of Obstetrics and Gynecology in Ruda Slaska, Medical University of Silesia, Ruda Slaska, Poland; ^2^Chair of Woman's Health, Medical University of Silesia, Katowice, Poland; ^3^Department of Obstetrics and Gynecology, Collegium Medicum, Nicolaus Copernicus University, Torun, Poland; ^4^Department of Obstetrics and Perinatology, Faculty of Health Sciences, Medical University of Warsaw, Warsaw, Poland

## Abstract

**Aim:**

This study aimed to investigate the cause of respiratory distress syndrome (RDS) in neonates from singleton pregnancies with preterm premature rupture of membranes (pPROM) between 24+0 and 36+6 weeks by using regression analysis for various factors.

**Methods:**

In 175 singleton pregnancies with pPROM, 95 cases of RDS (54,29%) were diagnosed. In all cases the following information was collected: latency period of PROM, gestational age at birth, Umbilical Artery Pulsatility Index (UA PI), Middle Cerebral Artery Pulsatility Index (MCA PI), fetal distress, antenatal steroids use, delivery type, pregnancy hypertension disease, gestational glucose intolerance or diabetes, neonatal laboratory parameters, gender, weight, Apgar score, and other neonatal complications. Logistic regression analysis was used to investigate the effect of variables on RDS.

**Results:**

The results of logistic regression analysis showed that the following variables are closely correlated with RDS: female gender (OR=0.52; 95%CI:0.28-0,97), antenatal steroids use (OR=0,46; 95%CI:0,34-0,64), abnormal UA PI and MCA PI (OR=2.96; 95%CI:1,43-6,12) (OR=2.05; 95%CI:1,07-3,95), fetal distress (OR=2.33; 95%CI:1,16-4,71), maternal HGB (OR=0.69; 95%CI:0,5-0,96), and neonatal RBC, HGB (OR=0.32; 95%CI:0,19-0,55) (OR=0.75; 95%CI:0,65-0,88).

**Conclusions:**

The main RDS risk factors in premature neonates are gender, abnormal fetoplacental circulation, and fetal distress. The laboratory parameters such as lower RBC and HGB count are observed in infants with RDS.

## 1. Introduction

Premature rupture of membranes (PROM) occurs in approximately 3-10% of all pregnancies; it is defined as a rupture of the membranes an hour before the start of uterine contractions, regardless of gestational age [1, 2]. Taking into account the gestational age, PROM is divided into two categories: before the 37th week of pregnancy defined as preterm premature rupture of membranes (pPROM) and after the 37th week of pregnancy referred to as term premature rupture of membranes (tPROM). pPROM complicates approximately 2-4% of singleton pregnancies and about 7-20% of multiple pregnancies [1, 2]. This complication is a significant cause of an increased morbidity and mortality for both infants and mothers [3, 4]. pPROM occurs among 30-40% of all preterm births, which is still a significant problem in perinatal medicine [5, 6]. Besides prematurity, neonatal complications include infection, sepsis, trauma, fetal distress, intraventricular hemorrhage, and respiratory distress syndrome [7, 8].

Respiratory distress syndrome (RDS) is one of the most common causes of neonatal respiratory failure and neonatal death. The underlying pathogenesis of RDS involves developmental immaturity of lungs, leading to inadequate pulmonary surfactant production [9]. It was previously believed that the most significant RDS factor is the prematurity. Despite many studies, the reason for the occurrence of RDS still remains unclear.

## 2. Objectives

This study aimed to investigate the cause of RDS in neonates from singleton pregnancies with pPROM between 24+0 and 36+6 weeks, using regression analysis for various factors, and thus provide a useful reference for its prediction.

## 3. Material and Methods

This investigation is a retrospective study approved by the bioethics committee of Silesian Medical University in Katowice, Poland. In the Department of Gynaecology and Obstetrics of the Municipal Hospital in Ruda Śląska from January 2011 to December 2014 a total of 175 singleton pregnancies with pPROM were hospitalized. A consecutive recruitment was used in this study.

The diagnosis of pPROM met the following criteria: (1) rupture of membranes based on the history, (2) leaking amniotic fluid found in physical examination, (2) singleton pregnancies between 24 + 0/7 and 36 + 6/7 weeks of gestation. Cases with dubious diagnosis were excluded.

In all cases the following information was collected: latency period of PROM; gestational age at birth; Umbilical Artery Pulsatility Index (UA PI); Middle Cerebral Artery Pulsatility Index (MCA PI); fetal distress; antenatal steroids use; maternal age at pregnancy, maternal haemoglobin (HGB), red blood cells (RBC), white blood cells (WBC) and platelets (PLT) count, maternal C-reactive protein (CRP) level, amniotic fluid index (AFI), and delivery mode; pregnancy hypertension disease; gestational glucose intolerance or diabetes; neonatal sex; weight; Apgar score at 1st, 3rd, 5th, and 10th minute; RBC, WBC, HGB, and PLT count; CRP level; and RDS, anaemia, congenital infection, and intraventricular haemorrhage (IVH).

In 95 cases (54,29%) RDS was diagnosed based on the following criteria: (1) acute onset; (2) representative clinical manifestations including progressive respiratory distress occurring shortly after birth, characteristic grunting respiration, retractions during inspiration, cyanosis, and reduced or absent breathing sounds; (3) typical chest x-ray findings, including hypoexpansion and diffuse, fine granular densities (grade I), air bronchograms caused by the atelectatic air sacs (grade II), ground-glass appearance (grade III), or white lungs caused by diffuse bilateral atelectasis (grade IV); (4) arterial blood gas analysis showing hypoxia, hypercapnia, and oxygen tension/fraction of inspired oxygen ratio (PaO2/FiO2) ≤ 26.7 kPa.

Other diagnostic criteria used in this study were [9–12] fetal distress as a significant abnormality in the fetal heart rate according to the result of fetal heart rate monitoring; congenital infection as fetal-neonatal infectious diseases such as pneumonia /septicemia caused by intra-amniotic infection; neonatal anaemia as HGB lower than 18 g/dl; IVH was diagnosed using transfontanel ultrasonography; all IVH grades were included in the study.

Logistic regression analysis was used to investigate the effect of variables on neonatal RDS. Univariate and multivariate logistic regression models were created. A p<0.05 was considered to be statistically significant.

## 4. Results

From 9657 deliveries in the Department of Gynaecology and Obstetrics of the Municipal Hospital in Ruda Śląska during the years 2011–2014, 175 cases (3,07%) met the pPROM criteria. RDS was diagnosed in 95 cases, which represents 54.29% of the studied group. The median latency period of pPROM was 19 hours and 48 minutes.

**Figure 1:**
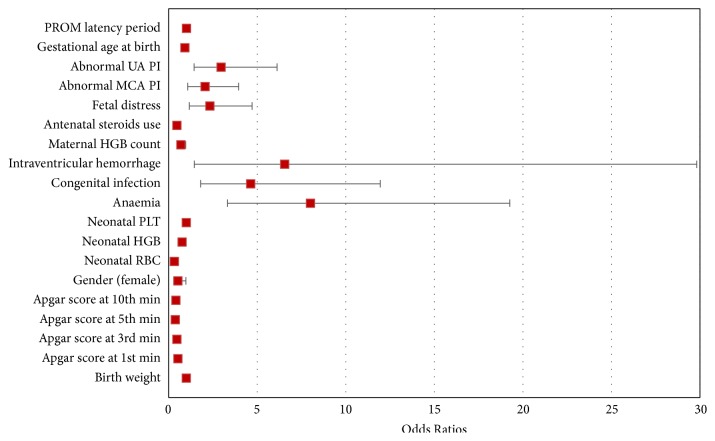
Odds ratios and confidence intervals for variables affecting the occurrence of preterm neonatal RDS–univariate logistic regression.

We found that the lower Apgar score at 1st, 3rd, 5th, and 10th minute (respectively, (OR = 0.52; 95% CI 0,4-0,68; p <0.001), (OR = 0, 46; 95% CI: 0,34-0,63; p <0.001), (OR = 0.37; 95% CI: 0,24-0,56; p <0.001), and (OR = 0.4; 95% CI: 0,26-0,6; p <0.001)); females sex (OR = 0.52; 95% CI: 0.28-0,97; p = 0.039); antenatal steroid use (OR = 0,46; 95% CI: 0,34-0,64; p <0.001); abnormal Umbilical Artery Pulsatility Index (UA PI) (OR = 2.96; 95% CI: 1,43-6,12; p = 0.003); abnormal Middle Cerebral Artery Pulsatility Index (MCA PI) (OR = 2.05; 95% CI: 1,07-3,95; p = 0.031); fetal distress (OR = 2.33; 95% CI: 1,16-4,71; p = 0.018); lower maternal HGB (OR = 0.69; 95% CI: 0,5-0,96; p = 0.025); and lower neonatal RBC and HGB (OR = 0.32; 95% CI: 0,19-0,55; p <0.001) and (OR = 0.75; 95% CI: 0,65-0,88; p <0.001) were the main risk factors of RDS in premature neonates (Table 1) (Figure 1).

A higher incidence of RDS resulted in newborns with anaemia (OR = 8; 95% CI: 3,32-19,26; p <0.001); congenital infection (OR = 4.63; 95% CI: 1,8-11,94; p =0.001); and intraventricular hemorrhage (OR = 6.55; 95% CI: 1,44-29,82; p = 0.015).

**Figure 2:**
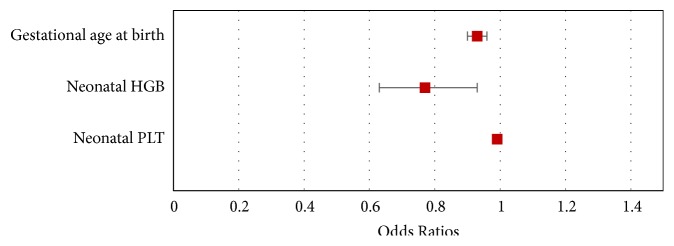
Odds ratios and confidence intervals for variables affecting the occurrence of preterm neonatal RDS-multivariate logistic regression.

In the analysis using multivariate logistic regression model, gestational age at birth (OR = 0.93; 95% CI 0,9-0,96; p <0.001), neonatal HGB (OR = 0.77; 95% CI: 0.63-0.93; p = 0.007), and neonatal PLT (OR = 0.9912; 95% CI: 0,9857-0,9967; p = 0.002) were the risk factors of RDS in premature neonates (Table 2) (Figure 2).

In this study variables such as delivery type; maternal and fetal WBC and CRP; maternal age; AFI; pregnancy hypertension disease; gestational glucose intolerance; or diabetes were not significant risk factors for RDS (p = ns) in preterm neonates.

## 5. Discussion

The occurrence of PROM, regardless of gestational age, is at level of 3-10% [1, 2]; 2-18% [13–15]. pPROM complicates approximately 2-4% of singleton pregnancies and 20-40% of all PROM cases [1, 2, 8, 13, 16]. In this study pPROM frequency was 3,07% which is similar to the one given in the literature.

According to Zanardo et al., RDS developed in 55.4% of the examined newborns from pregnancies complicated by pPROM [17], whereas JoonHo LEE et al. report that, in South Korea, the RDS was diagnosed in 47% of the cases [18]. In this study, RDS amounted 54.29% which is comparable to the percentages mentioned above.

The results of this study show that gender; antenatal steroid use; abnormal UA PI and MCA PI; fetal distress; and congenital infection are the main risk factors of RDS in preterm neonates from pPROM pregnancies.

This study shows that among female gender there is lower incidence of RDS in preterm neonates. The relative risk of RDS is 0,52 times lower for females than males. These data are confirmed in the literature [9, 19–21]. It was found that in gestation the female fetal lung produces surfactant earlier than the male one. The reasons for this may be as follows: (1) androgens delay lung fibroblast secretion of fibroblast-pneumocyte factor, which can delay the development of alveolar type II cells and reduce the release of surfactant; (2) androgens slow fetal lung development by adjusting the signalling pathways of epidermal growth factor and transforming growth factor-beta; (3) estrogen promotes the synthesis of phospholipids, lecithin, and surfactant proteins A and B; and (4) estrogen also improves fetal lung development by increasing the number of alveolar type II cells and by increasing the formation of lamellated bodies [9, 22–25].

Our study confirms that antenatal steroids' use reduces the risk for RDS. This fact results in the current international recommendations of the Royal College of Obstetricians and Gynaecologists (RCOG) in dealing with various accepted dosage schemes of corticosteroids.

Neonatal breathing disorders can be caused by circulatory system diseases. The main factors in this group are congenital heart disease, pulmonary hypertension, and congestive heart failure [26, 27]. No reports were found regarding fetoplacental circulation in relation to the development of neonatal RDS. However, the abnormal UA PI, MCA PI correlates with centralization of the cardiovascular system, which after the birth is an additional risk factor for RDS on the background of cardiovascular failure. Büke et al. concluded that pulmonary artery acceleration time to ejection time ratio (PATET) is a promising noninvasive tool to predict RDS in cases of preterm deliveries [28] while Laban M et al. find that measurement of fetal lung volume (FLV) or pulmonary artery resistance index (PA-RI) can help to predict RDS in preterm fetuses [29].

The results of this study show that congenital infection and fetal distress are strong RDS factors. A similar correlation was observed in many studies [9, 18, 19, 26, 30]. Fetal distress may lead to birth asphyxia. Asphyxia together with congenital infection causes the direct injury to the fetal lungs and alveolar type II cells, decreasing the synthesis and releasing surfactant [9, 31, 32]. Fetal-neonatal lung inflammation increases the permeability of the alveolar-capillary membrane to both fluid and solutes. This results in plasma proteins entering the alveolar hypophase, which further inhibits the function of surfactant [9, 31, 32].

In this study relationship between the lower count of RBC, HGB, PLT, and RDS was found. Correct levels of RBC, HGB, and PLT vary depending on the gestational age and prematurity; i.e., the less mature the newborn is, the lower the values are [33, 34]. Another factor affecting the RBC, HGB, and PLT values was the increased percentage of newborns with IUI and prolongation of PROM latency, who are characterized by significantly lower count of RBC, HGB, and PLT compared to noninfected newborns [34, 35].

There is also higher incidence of RDS in newborns affected by other complications such as anaemia, congenital infection, and intraventricular hemorrhage. This was also reflected in the literature [2, 13, 16, 31, 36]. Furthermore, in this study the occurrence of RDS was associated with lower PLT count; its deficiency leads to bleeding. Additional PLT reduction risk factors are prematurity and intrauterine infection [33]. This leads to the occurrence of both RDS and intraventricular hemorrhage [34].

## 6. Conclusions

The main risk factors of RDS in premature neonates are gender, abnormal fetoplacental circulation, and fetal distress. Other neonatal complications such as anaemia, congenital infection, and intraventricular haemorrhage increase the risk of RDS coexistence. The laboratory parameters abnormalities such as lower RBC, HGB, and PLT count are observed in infants with RDS.

## Figures and Tables

**Table 1:** Univariate logistic analysis of various factors for preterm neonatal RDS.

**Risk factor**	**Odds ratios**	**95**%** CI**	**p-value**	**Nr. of cases**
PROM latency period	1,0035	(1,0009;1,0061)	0,009	168
Gestational age at birth	0,9100	(0,88;0,94)	<0,001	170
Abnormal UA PI	2,9600	(1,43;6,12)	0,003	169
Abnormal MCA PI	2,0500	(1,07;3,95)	0,031	169
Fetal distress	2,3300	(1,16;4,71)	0,018	170
Antenatal steroids use	0,4600	(0,34;0,64)	<0,001	170
Maternal HGB	0,6900	(0,5;0,96)	0,025	153
Intraventricular hemorrhage	6,5500	(1,44;29,82)	0,015	167
Congenital infection	4,6300	(1,8;11,94)	0,001	169
Anaemia	8,0000	(3,32;19,26)	<0,001	168
Neonatal PLT	0,9916	(0,9871;0,9961)	<0,001	150
Neonatal HGB	0,7500	(0,65;0,88)	<0,001	150
Neonatal RBC	0,3200	(0,19;0,55)	<0,001	150
Gender (female)	0,5200	(0,28;0,97)	0,039	170
Apgar score at 10th min	0,4000	(0,26;0,6)	<0,001	168
Apgar score at 5th min	0,3700	(0,24;0,56)	<0,001	168
Apgar score at 3rd min	0,4600	(0,34;0,63)	<0,001	168
Apgar score at 1st min	0,5200	(0,4;0,68)	<0,001	168
Birth weight	0,9975	(0,9967;0,9983)	<0,001	170

**Table 2:** Multivariate logistic analysis of various factors for preterm neonatal RDS.

**Risk factor**	**Odds ratios**	**95**%** CI**	**p-value**	**Nr. of cases**
Gestational age at birth	0,9300	(0,9;0,96)	<0,001	150
Neonatal HGB	0,7700	(0,63;0,93)	0,007	150
Neonatal PLT	0,9912	(0,9857;0,9967)	0,002	150

## Data Availability

The data used to support the findings of this study are available from the corresponding author upon request.
